# Methylation in Predicting Progression of Untreated High-grade Cervical Intraepithelial Neoplasia

**DOI:** 10.1093/cid/ciz677

**Published:** 2019-07-25

**Authors:** Karolina Louvanto, Karoliina Aro, Belinda Nedjai, Ralf Bützow, Maija Jakobsson, Ilkka Kalliala, Joakim Dillner, Pekka Nieminen, Attila Lorincz

**Affiliations:** 1 Department of Obstetrics and Gynecology, Turku University Hospital, University of Turku, Turku, Finland; 2 Center for Cancer Prevention, Wolfson Institute of Preventive Medicine, Queen Mary University of London, London, United Kingdom; 3 Department of Obstetrics and Gynecology, Finland; 4 Department of Pathology, University of Helsinki and Helsinki University Hospital, Helsinki, Finland; 5 Department of Surgery & Cancer, Institute of Reproductive and Developmental Biology, Faculty of Medicine, Imperial College, London, United Kingdom; 6 Department of Laboratory Medicine, Karolinska Institute, Stockholm, Sweden

**Keywords:** DNA methylation, cervical intraepithelial neoplasia, CIN, high-grade squamous intraepithelial lesion, HSIL

## Abstract

**Background:**

There is no prognostic test to ascertain whether cervical intraepithelial neoplasias (CINs) regress or progress. The majority of CINs regress in young women, and treatments increase the risk of adverse pregnancy outcomes. We investigated the ability of a DNA methylation panel (the S5 classifier) to discriminate between outcomes among young women with untreated CIN grade 2 (CIN2).

**Methods:**

Baseline pyrosequencing methylation and human papillomavirus (HPV) genotyping assays were performed on cervical cells from 149 women with CIN2 in a 2-year cohort study of active surveillance.

**Results:**

Twenty-five lesions progressed to CIN grade 3 or worse, 88 regressed to less than CIN grade 1, and 36 persisted as CIN1/2. When cytology, HPV16/18 and HPV16/18/31/33 genotyping, and the S5 classifier were compared to outcomes, the S5 classifier was the strongest biomarker associated with regression vs progression. The S5 classifier alone or in combination with HPV16/18/31/33 genotyping also showed significantly increased sensitivity vs cytology when comparing regression vs persistence/progression. With both the S5 classifier and cytology set at a specificity of 38.6% (95% confidence interval [CI], 28.4–49.6), the sensitivity of the S5 classifier was significantly higher (83.6%; 95% CI, 71.9–91.8) than of cytology (62.3%; 95% CI, 49.0–74.4; *P* = 0.005). The highest area under the curve was 0.735 (95% CI, 0.621–0.849) in comparing regression vs progression with a combination of the S5 classifier and cytology, whereas HPV genotyping did not provide additional information.

**Conclusions:**

The S5 classifier shows high potential as a prognostic biomarker to identify progressive CIN2.

## Teaser

Many cervical intraepithelial neoplasias (CINs) regress spontaneously or persist, but to date there is no method to predict progression. In this study, we show that a DNA methylation classifier can predict progression or regression of untreated CIN grade 2.

**(See the Editorial Commentary by Del Mistro on pages 2591–2.)**

Cervical intraepithelial neoplasia (CIN) is caused by persistent human papillomavirus (HPV) infection, which is common in women of reproductive age. Most HPV infections and even CIN can regress without treatment [1, 2]. The mildest low-grade squamous intraepithelial lesions (LSILs, formerly CIN grade 1 [CIN1]) are treated with expectant management for up to 2 years before proceeding to local treatments in persistent disease [3]. Some guidelines also suggest this approach for CIN grade 2 (CIN2) in young women [3]. Recent evidence shows that 60% of CIN2 regress spontaneously within 2 years, while only 11% progress in women aged <30 years [4]. Overtreatment of any lesions should be avoided, especially in young women, as treatment significantly increases the risk of adverse outcomes in subsequent pregnancies [5–8].

To date, there is no prognostic test to ascertain whether a CIN lesion has a tendency to regress or progress, leaving treatment algorithms dependent on repeated examinations and testing. The HPV genotype does not appear to have enough predictive potential on the outcome. Although increasing proportions of severe lesions are caused by HPV16/18, the progressive potential remains mostly uncertain [9, 10]. Immunostaining of histological samples, for example with p16, shows increasing positivity with increasing severity of lesions but has not been found consistently prognostic [11–13].

DNA methylation of both HPV and host genes has been shown to increase with increasing severity of lesions [14–18]. Methylation as a screening triage to high-risk HPV (hrHPV)-positive women has also been found to be promising in predicting high-grade CIN (CIN2/3) [17, 19–21]. The usefulness of DNA methylation status in predicting the outcomes of prevalent cervical lesions has not been shown in a prospective longitudinal series. Here, we present results of the prognostic potential of a DNA methylation biomarker panel in a prospective cohort study of expectant management of untreated, histologically confirmed CIN2 in young women.

## METHODS

### Patients and Study Protocol

**Figure 1.**
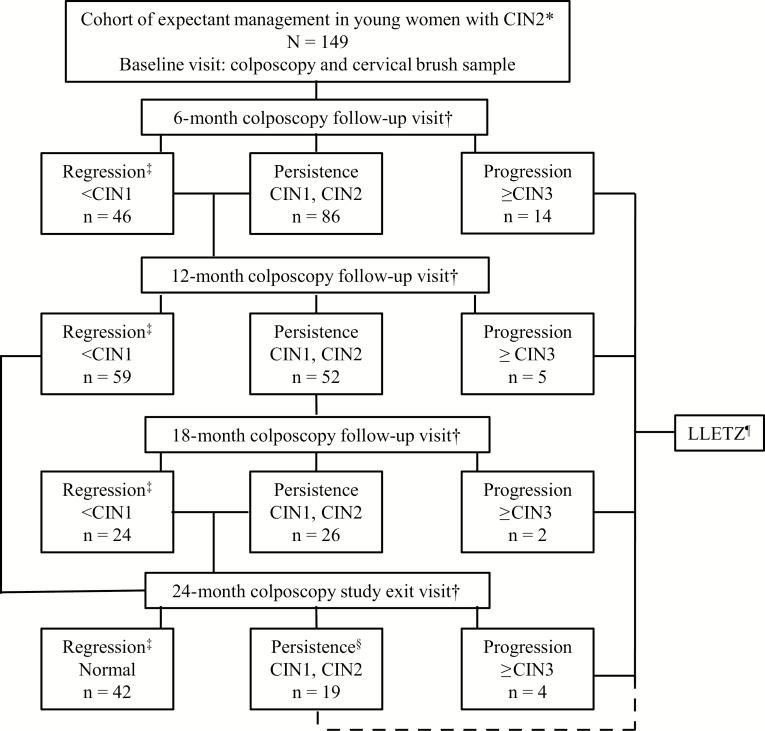
Flowchart of the expectant management of the cervical intraepithelial neoplasia (CIN) grade 2 study. The study started in the colposcopy clinic of Helsinki University Hospital, Finland, in September 2013, and is ongoing. The flowchart shows the numbers for the first 149 (age 18–30 years) women included in the current analyses who had a minimum of 2 follow-up visits completed as of November 2017. Women with follow-up diagnoses CIN grade 1 (<CIN1) were categorized as regression, women with CIN1 and/or CIN2 were categorized as persistence, and women with CIN grade 3 or worse (≥CIN3) were categorized as progression. Women with histological ≥CIN3 were treated with loop electrosurgical excision procedure, as were all women with a diagnosis (histological or colposcopic) of CIN1 or CIN2 after 2 years. Abbreviations: CIN, cervical intraepithelial neoplasia; LEEP, loop electrosurgical excision procedure; LLETZ, large loop excision of the transformation zone.

The study is part of an ongoing prospective cohort at the Colposcopy Unit of Helsinki University Hospital, Finland. Eligible women diagnosed with histological CIN2 were given written information on active surveillance as an alternative to the loop electrosurgical excision procedure (LEEP). The protocol, inclusion, and exclusion criteria of the study are shown in Figure 1.

At the baseline visit, after written consent, LEEP was not performed, but instead a new colposcopy was done to ensure eligibility. A cervical brush sample was obtained for HPV genotyping and methylation analyses. Scheduled study visits were at 6, 12, 18, and 24 months. LEEP was performed if histopathological progression to CIN3 or cancer (CIN3+) was observed at any visit or if persistence (CIN2 or CIN1) was observed at the 24-month visit. LEEP was also performed on patient request or if the patient moved out of the region. All histopathological and cytological samples were reviewed by the institution’s pathologists, and a second opinion was provided on all baseline biopsies by an expert pathologist (R. B.). If the histopathological diagnosis was not agreed upon, the patient was excluded from the study and treated according to Finnish treatment guidelines [22].

The study protocol was approved by the Helsinki University Hospital’s Ethical Committee and was registered in the International Standard Registered Clinical sTudy Number (ISRCTN) registry (ISRCTN91953024). Our study was conducted following REporting recommendations for tumour MARKer prognostic studies (REMARK) guidelines [23].

### Sample Processing and HPV Genotyping

At the baseline visit, the cells collected in sample transport medium (STM; Qiagen GMBH, Germany) were stored at −20°C. The samples were later divided into 3 aliquots and stored at −80°C. HPV genotyping was done at the Karolinska Institute, Stockholm, Sweden, with the Luminex assay as previously described [24].

### Methylation Analyses

DNA was extracted from aliquots of the STM with the QIAamp DNA Mini kit (Qiagen Inc, Hilden, Germany). Two hundred nanograms of DNA were used in the bisulfite conversion reactions, where unmethylated cytosines were converted to uracil with the EZ DNA-methylation kit (Zymo Research, Irvine, CA). Converted DNA from an equivalent of 1600 cells per sample were amplified by methylation‐independent polymerase chain reaction primers, and the amplicons were tested by pyrosequencing for DNA methylation of *EPB41L3* and the late (L1 and/or L2) regions of HPV16, HPV18, HPV31, and HPV33, as previously described [19, 25, 26]. The laboratory was blinded to HPV genotyping results; therefore, each methylation assay in the S5 classifier was run on all specimens. No HPV type inconsistencies were detected on the methylation vs the HPV genotyping results.

### Statistical Analyses

The primary clinical contrast was progression to CIN3+ vs regression to <CIN1. We also evaluated the former 2 vs persistence (CIN2 or conversion to persistent CIN1). Only women whose histopathological diagnoses changed to <CIN1 and remained constant during all follow-up visits were considered as regressed. If a woman’s normal histology subsequently progressed back to CIN1 or CIN2, she was categorized as persistent for CIN. Our primary question was whether the S5 classifier or the different methylation biomarkers within the classifier could predict progression to CIN3+ among women with CIN2. The clinical outcome groups were defined according to the histopathological findings during follow-up. The S5 classifier was defined as S5 classifier = 30.9(EPB41L3) + 13.7(HPV16L1) + 4.3(HPV16L2) + 8.4(HPV18L2) + 22.4(HPV31L1) + 20.3(HPV33L2) with individual CpG sites as described previously [21, 27]. DNA methylation status for baseline missing values (n = 8) of HPV16 were imputed with the value of zero for any HPV16-negative sample (n = 5) and by the median for HPV16-positive samples (n = 3). Missing values for *EPB41L3* (n = 8) were imputed by the median independently of their HPV infection status. Eight women with missing HPV genotyping results were imputed as HPV-negative. Our main measures of performance were odds ratios (OR), sensitivity, and specificity comparisons, with cutoffs for the S5 classifier set at the upper tertile (upper one-third of methylation levels) or at the predefined and validated cut-point of the S5 classifier = 0.8. Cytology was categorized according to the Bethesda classification. HPV16, HPV18, HPV31, and HPV33 combinations were regarded as binary variables, with any of these types detected regarded as a positive result vs an all HPV-negative result [19, 27].

The differences between baseline characteristics and mean methylation levels in the 3 clinical outcome groups were compared with the Mann-Whitney or Fisher exact test or nonparametric test for trend, as applicable. The Cuzick test for trend was used to compare the mean methylation levels of the different markers among the diagnostic groups. Unconditional logistic regression ORs and 95% confidence intervals (CIs) were used to evaluate the associations of mean methylation level or the upper tertile level of different methylation markers and various clinical outcome comparisons. The high tertile of methylation was defined as any value within the upper one-third of the distribution of methylation values identified for each methylation biomarker in the specific outcome category comparison [14]. Multivariable models of logistic regression were used to evaluate possible confounding factors in the methylation vs clinical outcome comparisons and to investigate different biomarker associations between the various clinical outcome groups.

The difference in sensitivity at a selected methylation test cut-point, where the specificity of the methylation test was held equal to the reference comparator (cytology, HPV16/18 genotyping, or both), was assessed using the McNemar test. The performance of different methylation markers and screening protocols was measured by receiver operating characteristic analysis by comparing the area under the curve (AUC). Kaplan-Meier curves were used to assess the cumulative proportions of women who progressed to CIN3+ by time (in months) since the diagnosis of CIN2. In this analysis, persistent CIN1/2 was regarded as nonprogression. A likelihood ratio test was used to assess differences between women with all positive biomarkers to women who tested negative for all markers. Cox proportional hazards regression models were used to estimate unadjusted hazard ratios (HRs) (95% CI) in order to examine associations between median methylation and CIN2 progression (date of CIN3+ diagnosis). All *P* values were 2 sided, and *P < *.05 was regarded as significant. Statistical analyses were performed using Stata15 (Stata Corp., College Station, TX).

## RESULTS

A total of 149 women with histologically confirmed CIN2 and at least 2 (6 monthly) follow-up visits were included. A flowchart of the study is shown in Figure 1. Of the 149 women, 147 had a follow-up visit at 6 months (2 women had the first follow-up visit at month 12). A total of 116 women had 2 follow-up visits (at 6 and 12 months); 52 women had an 18-month visit, and 65 women completed the full schedule of follow-up visits to 24 months. All 25 women (17%) who progressed to CIN3+ were treated using LEEP. Of 88 women (59%) categorized as regressed to <CIN1, 42 exited the study without treatment, and the remaining women are still under follow-up. Of the 36 women (24%) categorized as persistent with CIN1/2, 7 LEEP procedures were performed at the end of the 24-month period, and all had histological CIN2. The remaining 12 women in the persistent group at the 24-month visit had CIN1 and are being followed up according to clinical guidelines. The remaining 17 women in the persistent group have not yet completed all follow-up visits.

The baseline characteristics of the women are presented in Table 1. The mean age was 26 years (range, 25.9–27.0 years) and did not differ significantly between the 3 outcome groups. Twenty-one of the 25 women who progressed to CIN3 were hrHPV (1 or more of types 16, 18, 31, 33, 35, 39, 45, 51, 52, 56, 58, 59)-positive. In contrast, 63 of the 88 women who regressed to <CIN1 were hrHPV-positive. Finally, 32 of the 36 women who persisted as CIN1/2 were hrHPV-positive. Overall, 82.3% (116/141) of the women were positive for hrHPV. Of these, 52.6% (61/116) were positive for HPV16, while 9.5% (11/116) were positive for HPV18. There was a significant difference (*P* = .02) between the regression and persistence groups in terms of hrHPV positivity; 94.1% of women who persisted were positive compared to 75.0% in the regression group.

**Table 1.** Baseline Characteristics of the 149 Women from the Cohort Study on Expectant Management of Cervical Intraepithelial Neoplasia Grade 2

Characteristic	All Women	Regression	Persistence	Progression
	(N = 149)	(n = 88)	(n = 36)	(n = 25)
Age (mean), y	26.0	25.9	25.3	27.0
Smoking (n = 128)				
No	61	41	8	11
Yes	67	37	23	7
Cigarettes per day (mean)	5.4	5.8	4.7	5.9
Papanicolaou cytology				
No intraepithelial lesion or malignancy	23	15	7	1
Greater than or equal to atypical squamous cells of undetermined significance	126	73	29	24
Any HPV (n = 141)				
Negative	25	21	2	2
Positive	116	63	32	21
HPV16+	61	32	15	14
HPV18+	11	4	5	2
HPV31+	19	8	7	4
HPV33+	8	4	3	1

All recruited women had a biopsy confirmed cervical intraepithelial neoplasia grade 2 (CIN2) histology at the baseline of the study. The women were divided into 3 clinical outcome groups (regression <CIN1, persistence CIN1/2, and progression ≥CIN3) according to the histological findings during the study follow-up. Significant differences (Mann-Whitney or Fisher exact test or nonparametric test for trend) between the baseline characteristics among women in the 3 outcome groups was only detected with any HPV status with regression vs persistence (*P* = .02) comparison.

Abbreviation: HPV, human papillomavirus.

Mean methylation levels of the host gene *EPB41L3* (CpG sites 438, 427, 425), the viral HPV16L1-gene (CpG sites 6367, 6389), and the S5 classifier according to clinical outcome are presented in Supplementary Figure 1. Statistical significance in pairwise comparisons of progression to CIN3+ vs regression to <CIN1 was found with *EPB41L3* alone (*P* = .02), while for the full S5 classifier, the difference was highly significant (*P* = .001).

Table 2 presents results for clinical outcome comparisons between each individual outcome (regression, progression, persistence, and combinations of the latter with the former 2) for the S5 classifier, *EPB41L3*, and the HPV16L1 methylation biomarkers, using either the prevalidated high tertile levels or the median methylation as cutoffs [14]. For the high tertile cutoff, the S5 classifier reached statistical significance in almost all comparisons (except persistence vs progression), with the highest OR of 4.84 (95% CI, 1.35–17.41) and an AUC of 0.718 (95% CI, 0.61–0.83) observed for regression vs progression. ORs for mean methylation were found to be significant in all clinical outcome comparisons except regression vs persistence for the S5 classifier and with *EPB41L3* alone in all except the intermediate group comparisons of persistence and regression or progression. HPV16L1 methylation alone did not show a significant association with any of the comparisons.

**Table 2.** Odd Ratios for the Association Between Different Methylation Biomarkers and Clinical Outcome Comparisons

Clinical Outcome Comparison	Methylation Marker	OR1 (95% CI)^a^	OR2 (95% CI)	Area Under the Receiver Operator Characteristic Curve (95% CI)
Regression vs persistence	*EPB41L3*	1.03 (.46, 2.35)	1.06 (.95, 1.18)	0.518 (.41, .63)
	HPV16L1	1.21 (.55, 2.65)	0.99 (.97, 1.01)	0.497 (.40, .59)
	S5 classifier	**2.61 (1.03, 6.61)**	1.04 (.95, 1.14)	0.567 (.46, .68)
Regression vs progression	*EPB41L3*	2.29 (.78, 6.68)	**1.14 (1.03, 1.26)**	**0.649 (.52, .77)**
	HPV16L1	1.92 (.79, 4.73)	1.01 (.99, 1.03)	0.576 (.46, .69)
	S5 classifier	**4.84 (1.35, 17.41)**	**1.17 (1.06, 1.30)**	**0.718 (.61, .83)**
Persistence vs progression	*EPB41L3*	2.55 (.78, 8.34)	1.08 (.99, 1.19)	**0.639 (.50, .78)**
	HPV16L1	1.59 (.57, 1.44)	**1.03 (1.00, 1.07)**	0.588 (.45, .73)
	S5 classifier	2.86 (.88, 9.33)	**1.15 (1.01, 1.30)**	**0.676 (.54, .82)**
Regression/persistence vs progression	*EPB41L3*	2.28 (.80, 6.49)	**1.12 (1.03, 1.21)**	**0.646 (.52, .77)**
	HPV16L1	1.82 (.77, 4.33)	1.01 (.99, 1.03)	0.580 (.46, .70)
	S5 classifier	**4.48 (1.27, 15.77)**	**1.16 (1.06, 1.28)**	**0.706 (.60, .81)**
Regression vs persistence/progression	*EPB41L3*	1.37 (.68, 2.75)	**1.09 (1.01, 1.19)**	0.572 (.48, .67)
	HPV16L1	1.47 (.76, 2.83)	1.00 (.98, 1.02)	0.530 (.44, .62)
	S5 classifier	**2.68 (1.27, 5.64)**	**1.10 (1.02, 1.19)**	**0.630 (.54, .72)**

Univariable ORs with 95% CIs for the associations between the upper tertile^a^ OR1 and mean methylation, OR2 levels of the host gene *EPB41L3* (CpG 438, 427, 425) and viral human papillomavirus (HPV) 16 L1 gene (CpG 6367, 6389), and the S5 classifier (>0.8 cutoff) and the different clinical outcome comparisons. The last column shows the area under the curve derived from receiver operating characteristic analysis of the diagnostic performance of mean methylation cutoffs of *EPB41L3*, the viral HPV16 L1 gene, and the >0.8 cutoff for the S5 classifier with the different clinical outcome comparisons. Significant ORs are shown in bold.

Abbreviations: CI, confidence interval; OR, odds ratio.

^a^ In OR1, a high tertile level was defined as one-third of the upper methylation levels that was identified in each of the outcome category comparisons.

We explored the performance of a previously validated high tertile cutoff for the S5 classifier and compared this variable to a cytology cut-point of less than or equal to the atypical squamous cells of undetermined significance (ASC-US) vs ≥LSIL and to HPV16/18 and HPV16/18/31/33 positive vs negative (with regression as the referent group; Table 3). The S5 classifier showed the highest significant association with a progression OR of 3.39 (95% CI, 1.35–8.50) followed by HPV16/18/31/33 genotyping with an OR of 3.17 (95% CI, 1.15–8.68). HPV16/18/31/33 genotyping also had a significant association with CIN2 persistence that the other markers did not show, giving an OR of 3.50 (95% CI, 1.44–8.52).

**Table 3.** Odds Ratios for the Association Between the Different Clinical Outcome Comparisons and the Different Markers

Clinical Outcome	Odds Ratio (95% Confidence Interval)			
	S5 Classifier	Papanicolaou Cytology Less than or Equal to Atypical Squamous Cells of Undetermined Significance vs Greater than or Equal to Low-grade Squamous Intraepithelial Lesion	HPV16/18 Genotyping	HPV16/18/31/33 Genotyping
Regression	1.00	1.00	1.00	1.00
Persistence	1.33 (.58, 3.07)	1.00 (.43, 2.33)	1.99 (.91, 4.35)	**3.50 (1.44, 8.52)**
Progression	**3.39 (1.35, 8.50)**	2.32 (.73, 7.42)	2.38 (.96, 5.91)	**3.17 (1.15, 8.68)**

Comparison of the upper tertile^a^ level of the S5 classifier, the Papanicolaou cytology comparison of less than or equal to atypical squamous cells of undetermined significance vs greater than or equal to low-grade squamous intraepithelial lesion, the HPV16/18, and the HPV16/18/31/33 genotyping positive or negative. The S5 classifier and HPV16/18/31/33 genotyping were the 2 significant prognostic variables, shown in bold.

Abbreviation: HPV, human papillomavirus.

^a^A high tertile level was defined as one-third of the upper methylation levels of the S5 classifier at baseline.

In a multivariable model comparing ORs of *EPB41L3* and the S5 classifier to different clinical outcomes, only the S5 classifier was shown to be an independent predictor of outcomes among the regression vs persistence and regression/persistence vs progression groups (adjusted for HPV16/18/31/33 status, abnormal cytology, smoking status, and age; Supplementary Table 1).

**Figure 2.**
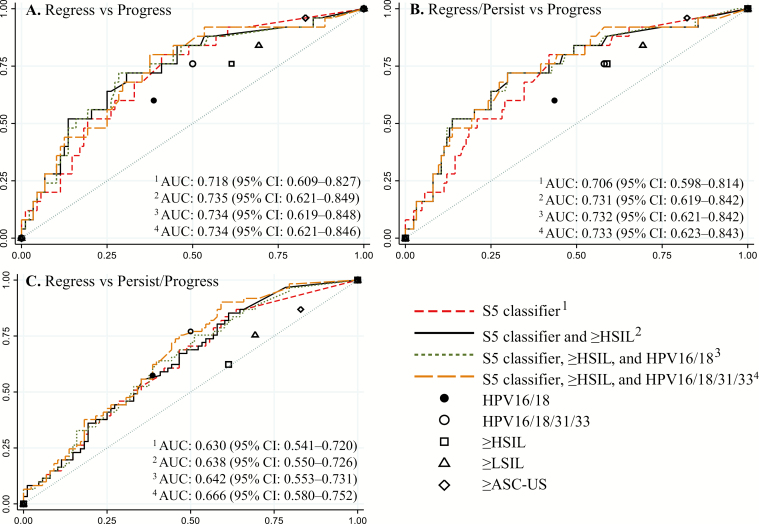
Receiver operating characteristic curves for the performance of the S5 classifier and in combination with other tests. The S5 classifier performance alone and in combination with ≥HSIL cytology and/or HPV16/18 or HPV16/18/31/33 genotyping positivity tested in different clinical outcome categories. The S5 classifier alone (red dashed line, AUC 1), the S5 classifier combined with ≥HSIL (black solid line, AUC 2), the S5 classifier combined with ≥HSIL and HPV16/18 (green dotted line, AUC 3), or the S5 classifier combined with ≥HSIL and HPV16/18/31/33 (orange dash line, AUC 4) in the following clinical outcome categories: (*A*) regression vs progression, (*B*) regression/persistence vs progression, and (*C*) regression vs persistence/progression. For each clinical outcome category (*A*–*C*), the corresponding performance of the single tests, HPV16/18 genotyping (black solid circle), HPV16/18/31/33 genotyping (hollow black circle), and the different cytological abnormality endpoints, are shown; ≥ASC-US (hollow diamond), ≥LSIL (hollow triangle), and ≥HSIL (hollow square). Abbreviations: ASC-US, atypical squamous cells of undetermined significance; AUC, area under the receiver operating characteristic curve; CI, confidence interval; HPV, human papillomavirus; HSIL, high-grade squamous intraepithelial lesion; LSIL, low-grade squamous intraepithelial lesion.

Sensitivity, specificity, and positive and negative predictive values were compared between reference tests (cytology at varying cut-points and HPV16/18 positivity or negativity) and the index methylation marker *EPB41L3* alone and the S5 classifier in the outcome of regression vs persistence/progression (Supplementary Table 2). Our comparisons focused on sensitivity differences when the index test cutoffs were set to allow the closest approximation of specificity between the index and the comparator reference test. The S5 classifier showed significantly increased sensitivity compared to cytology (cut-point ≤ASC-US vs ≥LSIL) with a sensitivity of 86.9% (95% CI, 75.8–94.2) for the S5 classifier vs 75.4% (95% CI, 62.7–85.5) for cytology ≥LSIL (*P* = .05). In contrast, a cytology cut-point of negative for intraepithelial lesion or malignancy vs ≥ASC-US was essentially nonspecific for progression, thereby producing an unrealistic comparison to the S5 classifier. With a cytology cut-point at ≤LSIL vs high-grade squamous intraepithelial lesion or worse (≥HSIL) and a set specificity of 38.6% (95% CI, 28.4–49.6), the sensitivities were significantly different at 83.6% (95% CI, 71.9–91.8) for the S5 classifier and 62.3% (95% CI, 49.0–74.4) for cytology (*P* = .005). HPV16/18 genotyping performed similarly to the S5 classifier when specificity of both were made equal (*P* = 1.00). However, this comparison produced a maximum sensitivity of 57%, which we regard as too low for a prognostic biomarker. Indeed, the S5 classifier could be set to a much higher sensitivity (>75%) albeit with a commensurate loss in specificity compared to HPV16/18 genotyping (Figure 2). HPV16L1 methylation and HPV16 genotyping markers were also tested but showed a much lower diagnostic utility compared to *EPB41L3* and the S5 classifier among the clinical outcome comparison of regression vs persistence/progression.

The performance of the S5 classifier alone and in combination with other tests (≥HSIL cytology and/or HPV16/18- or 16/18/31/33-positivity) was tested in different clinical outcome categories either separately or grouped (Figure 2). The highest AUC was 0.735 (95% CI, 0.621–0.849) in the regression vs progression clinical outcome comparison, with a combination of the S5 classifier above a cutoff of 0.8 and cytology ≥HSIL regarded as positive. Combining HPV16/18- or HPV16/18/31/33-positives with the S5 classifier and cytology did not provide any additional advantage. This was seen in all clinical outcome comparison groups, except with the regression vs persistence/progression group where combining HPV16/18/31/33 with the S5 classifier gave the highest AUC of 0.666 (95% CI, 0.580–0.752). Comparisons of *EPB41L3*, the S5 classifier, and HPV16-positivity in clinical outcome comparisons of persistence vs progression and regression vs persistence/progression are presented in Supplementary Figure 2. It is noteworthy that the S5 classifier alone provided better performance in discriminating the clinical outcome of progression vs regression, whereas HPV16/18/31/33-positivity performed better in predicting persistent HPV infection.

**Figure 3.**
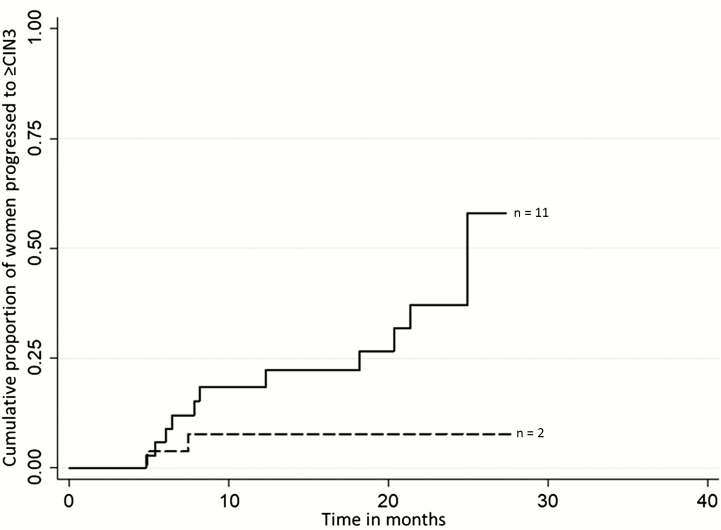
Cumulative proportions of women who progressed to ≥CIN grade 3 by time since the diagnosis of CIN2. In this analysis, persistent CIN1 or CIN2 were regarded as nonprogressions. The graph shows the distribution by time (in months) of women positive for the following: S5 classifier, human papillomavirus (HPV)16/18 and cytology greater than or equal to high-grade squamous intraepithelial lesion (HSIL; solid line) vs women who were negative for all of these markers: the S5 classifier ≤0.8, HPV16/18 negative, and Papanicolaou smear <HSIL (dashed line). There was a significant difference between these predictors (Likehood-ratio [LR] test *P* = .03). Abbreviation: CIN, cervical intraepithelial neoplasia.

Figure 3 shows a significant difference (Likehood-ratio [LR] test, *P* = .03) between cumulative proportions of progression to CIN3+ distributed by time in women positive for the S5 classifier, HPV16/18, and cytology ≥HSIL vs negative for all the previous tests (S5 classifier ≤0.8, HPV16/18-negative, and cytology <HSIL). Cox proportional hazards regression modeling was used to estimate the HR in order to examine associations between median methylation of the S5 classifier and CIN2 progression (date of the CIN3+ diagnosis) among the groups of regression/persistence vs progression. The HR for the S5 classifier alone was 4.19 (95% CI, 1.57–11.17), and the HR was 3.84 (95% CI, 1.13–13.04) when adjusted for ≥HSIL cytology, HPV16/18-positivity, age, and smoking status.

## DISCUSSION

This is the first study to assess the predictive potential of the S5 classifier in a prospective longitudinal series of patients with histological CIN2 at baseline. We found the S5 classifier to be a significant predictor of progression vs regression in women with untreated CIN2, even after adjusting for cytology, HPV16/18/31/33 genotyping, age, and cigarette smoking. *EPB41L3* and HPV16 L1 individually did not perform similarly, with the former biomarker being much better than the latter for progression as the main outcome. The sensitivity of the S5 classifier was significantly higher than of cytology with varying cutoffs (≤ASC-US vs ≥LSIL and ≤LSIL vs ≥HSIL) in assessment of clinical outcomes of regression vs persistence/progression. With a high tertile cutoff value for methylation, the ORs in favor of the S5 classifier prognostic potential were even higher, and the greatest AUC 0.735 (95% CI, 0.621–0.849) was achieved when the S5 classifier was combined with ≥HSIL cytology.

Although HPV16/18/31/33 genotyping was as good as the S5 classifier in predicting regression vs the combination of persistence/progression (Figure 2C), it was not as good as the S5 classifier in predicting progression vs regression (Figure 2A and Table 3). The equivalence of the S5 classifier to HPV16/18/31/33 prediction for the combination of persistence and progression categories appears to be driven mainly by the relatively larger persistence group. It should be considered that the natural history of long-term HPV persistence with respect to eventual true progression of CIN2 to CIN3+ vs regression to normal beyond 2 years remains unclear. In our clinical setting, persistence of CIN1/2 for 2 years was taken as an indication for treatment; however, we do not know what proportion of these treatments were really necessary to prevent cervical cancer.

We have a unique study population and CIN2 management strategy. The strengths of our study include focus on a very important clinical component, that is, the ability to conduct vigilant follow-up by an expert medical team and expert histopathological diagnosis of CIN categories. We also have a careful parallel comparison of the different methylation panels and other comparison tests to minimize bias. A weakness is that our results cannot be directly generalized to other histopathological diagnoses. Also, our study was restricted to young women and the length of follow-up varied, which may result in some reclassification of outcomes (regression, persistence) as follow-up continues. This was especially the case regarding those in the persistent category as the true nature of their disease remains undefined.

The S5 classifier has previously been proven to identify the risk of ≥CIN2/3 in hrHPV-positive women in cross-sectional studies based on screening and colposcopy populations where the classifier outperformed triage with HPV16/18 genotyping [19, 27]. In the current study, we show that the S5 classifier can differentiate between regressive and progressive CIN2. Methylation of other combinations of host and HPV genes have also been found to increase proportionally with severity of lesions [17]. However, this has not been examined in a longitudinal series of patients with CIN, except for a small series of HIV-positive women where patients with persistent CIN2/3 had higher methylation of *EPB41L3* than women with <CIN1 or regression to <CIN1 [28]. Another host gene *FAM19A4* has been shown to more often be methylation-positive in high-grade disease if the hrHPV-infection had persisted longer [16].

Ours is the first study to show significant differences in methylation within the uniform histological diagnosis of CIN2, the outcome of which is highly variable and depends on the intrinsic progressive or regressive potential as well as clinical management philosophy. We reveal a new utility of the S5 classifier DNA methylation measurement, specifically as a classifier for assessing risk of progression in histologically confirmed CIN2.

A prognostic test for CIN could greatly alter treatment algorithms. A well-recognized dilemma of expectant management strategies is the great intra- and interobserver variability in both cytological and histological diagnoses [29–31]. This results in misclassification of lesions, multiple follow-up visits, and either delayed or premature treatment, exacerbating the potential harm to the patient. An improved predictive test could revolutionize management of CIN2 as cases with progressive potential could be treated sooner and regressive cases managed expectantly, with persistent CIN2 perhaps eventually also going untreated for longer periods to allow more regressions. Additional studies on predicting the risk of progression from CIN3 to invasive cancer are also warranted. Clearly, the biological and clinical meaning of CIN2 persistence remains a major issue.

## Supplementary Data

Supplementary materials are available at *Clinical Infectious Diseases* online. Consisting of data provided by the authors to benefit the reader, the posted materials are not copyedited and are the sole responsibility of the authors, so questions or comments should be addressed to the corresponding author.

ciz677_suppl_Supplementary_Information

## Notes

***Financial support.*** This work was supported by the Sigrid Jusélius Foundation, Finnish Medical Foundation, Helsinki and Uusimaa Hospital District, Cancer Foundation (Finland), and Cancer Research UK (grant C569/A10404).

***Potential conflicts of interest.*** J. D. has received grants from Roche and from Genomica outside the submitted work. All other authors report no potential conflicts. The author has submitted the ICMJE Form for Disclosure of Potential Conflicts of Interest. Conflicts that the editors consider relevant to the content of the manuscript have been disclosed.
