# Supplementary material for: Methylation in Predicting Progression of Untreated High-grade Cervical Intraepithelial Neoplasia
Source: Clin Infect Dis. 2019 Jul 25;70(12):2582–90. doi: 10.1093/cid/ciz677 (PMC7286376; doi:10.1093/cid/ciz677)
Supplement: ciz677_suppl_Supplementary_Information [file ciz677_suppl_supplementary_information.docx]

**Supplementary Material**

| **Supplementary Table 1.** **Multivariate model of odd ratios (OR, and 95 % confidence interval) of the mean methylation of the host gene *EBP41L3* (CpG 438, 427, 425) and the S5-Classifier compared to different clinical outcomes**. S5-Classifier shows to be independent predictors of clinical outcomes with no confounders found among the regress vs. progress and regress/persist vs progress group. Significant differences are shown in bold. | | | | | | | |
| --- | --- | --- | --- | --- | --- | --- | --- |
| **Clinical outcome comparison** | **Methylation marker** | **Crude OR (95% CI)** | **OR (95% CI)^1^** | **OR (95% CI)^2^** | **OR (95% CI)^3^** | **OR (95% CI)^4^** | **OR (95% CI)^5^** |
| Regress vs. Progress | EPB41L3 | **1.14 (1.03, 1.26)** | 1.11 (0.99, 1.24) | **1.13 (1.01, 1.26)** | **1.15 (1.03, 1.29)** | **1.16 (1.04, 1.29)** | **1.13 (1.02, 1.25)** |
|  | S5-Classifier | **1.17 (1.06, 1.30)** | **1.12 (1.00, 1.27)** | **1.14 (1.01, 1.28)** | **1.16 (1.04, 1.29)** | **1.18 (1.06, 1.31)** | **1.15 (1.03, 1.28)** |
| Regress/Persist vs. Progress | EPB41L3 | **1.12 (1.03, 1.21)** | 1.09 (0.99, 1.19) | **1.11 (1.01, 1.21)** | **1.12 (1.02, 1.22)** | **1.12 (1.03, 1.22)** | **1.11 (1.03, 1.21)** |
|  | S5-Classifier | **1.16 (1.06, 1.28)** | **1.14 (1.02, 1.28)** | **1.16 (1.03, 1.30)** | **1.16 (1.05, 1.29)** | **1.17 (1.07, 1.29)** | **1.15 (1.05, 1.27)** |
| Regress vs. Persist/Progress | EPB41L3 | **1.09 (1.01, 1.19)** | 1.08 (0.99, 1.18) | 1.08 (0.99, 1.17) | **1.09 (1.00, 1.18)** | **1.09 (1.01, 1.19)** | **1.09 (1.00, 1.18)** |
|  | S5-Classifier | **1.10 (1.02-1.19)** | 1.04 (0.95, 1.13) | 1.04 (0.95, 1.13) | 1.05 (0.98, 1.15) | **1.10 (1.02, 1.19)** | 1.06 (0.98, 1.15) |
| ^1^adjusted by age, smoking status, abnormal cytology and HPV16/18/31/33 status  ^2^adjusted by smoking status, abnormal cytology and HPV16/18/31/33 status  ^3^adjusted by abnormal cytology and HPV16/18/31/33 status  ^4^adjusted by abnormal cytology  ^5^adjusted by HPV16/18/31/33 status | | | | | | | |

| **Supplementary Table 2. Sensitivity, specificity, positive and negative predictive values of comparisons between the reference tests: 1) Papanicolaou cytology at different cut-offs, and 2) HPV 16/18 genotyping positive or negative, versus the index methylation biomarker tests: 3) *EPB41L3* and 4) S5-classifier.** Performance values for various cut-offs of 1) cytology and 2) HPV genotyping were calculated from dichotomous cut-offs as shown below. The index methylation biomarker comparisons 3) and 4) to the reference tests focused on sensitivity differences when the index test cut-offs were set to allow the closest approximation of specificity between the index and the reference tests.* At these chosen cut-off levels, the sensitivity values are shown below and were compared for significance with the McNemar’s test, using as the clinical outcome regression vs. persistence/progression. | | | | | |
| --- | --- | --- | --- | --- | --- |
| **Compared markers and cut-offs** | **Specificity^†^**  **% (95% CI)** | **Sensitivity**  **% (95% CI)** | **PPV**  **% (95% CI)** | **NPV**  **% (95% CI)** | **McNemar test for sensitivity**  ***p-value*** |
| Pap cytology: NILM vs ≥ASC-US | 17.0 (9.9, 26.6) | 86.9 (75.8, 94.2) | 42.1 (33.3, 51.2) | 65.2 (42.7, 83.6) | Ref |
| *EPB41L3* | 31.8 (22.3, 42.6) | 80.3 (68.2, 59.4) | 45.0 (35.4, 54.8) | 70.0 (53.5, 83.4) | NC |
| S5-Classifier | 17.0 (9.9, 26.6) | 90.2 (79.8, 96.3) | 43.0 (34.3, 52.0) | 71.4 (4.8, 88.7) | 0.69 |
| Pap cytology: ≤ASC to US vs ≥LSIL | 30.7 (21.3, 41.4) | 75.4 (62.7, 85.5) | 43.0 (33.5, 52.9) | 64.3 (48.0, 78.4) | Ref |
| *EPB41L3* | 31.8 (22.3, 42.6) | 80.3 (68.2, 89.4) | 45.0 (35.4, 54.8) | 70.0 (53.5, 83.4) | 0.41 |
| S5-Classifier | 31.8 (22.3, 42.6) | 86.9 (75.8, 94.2) | 46.9 (37.5, 56.5) | 77.8 (60.8, 89.9) | **0.05** |
| Pap cytology: ≤LSIL vs ≥HSIL | 38.6 (28.4, 49.6) | 62.3 (49.0, 74.4) | 41.3 (31.1, 52.1) | 59.6 (45.8, 72.4) | Ref |
| *EPB41L3* | 38.6 (28.4, 49.6) | 70.5 (57.4, 81.5) | 44.3 (34.2, 54.8) | 65.4 (50.9, 78.0) | 0.28 |
| S5-Classifier | 38.6 (28.4, 49.6) | 83.6 (71.9, 91.8) | 48.6 (38.7, 58.5) | 77.3 (62.2, 88.5) | **0.005** |
| HPV16/18 genotyping pos vs neg | 61.4 (50.4, 71.6) | 57.4 (44.1, 70.0) | 50.7 (38.4, 63.0) | 67.5 (56.1, 77.6) | Ref |
| *EPB41L3* | 61.4 (50.4, 71.6) | 42.6 (30.0, 55.9) | 43.3 (30.6, 56.8) | 60.7 (49.7, 70.9) | 0.18 |
| S5-Classifier | 59.1 (48.1, 69.5) | 57.4 (44.1, 70.0) | 49.3 (37.2, 61.4) | 66.7 (55.1, 76.9) | 1.00 |
| *HPV16 L1 methylation and HPV16 genotyping markers where also tested but showed a much lower diagnostic utility compared to the *EPB41L3* and S5-Classifier among the clinical outcome comparison of regression vs. persistence/progression. HPV16 L1 methylation had specificity of 36.4% (95%CI 26.4, 47.3) and sensitivity of 54.1% (95%CI 40.8, 66.9); and HPV16 genotyping had specificity of 61.9% (95%CI 50.7, 72.3) and sensitivity 50.9% (95%CI 37.3, 64.4).  ^†^ Empirically assessed at the threshold that yielded the closest specificity to the cytology comparison or HPV16/18 genotyping.  Abbreviations: Pap: Papanicolaou, NILM: no intraepithelial lesion or malignancy, ASC-US: atypical squamous cells of undetermined significance, LSIL: low grade squamous intraepithelial lesion, HSIL: high grade squamous intraepithelial lesion, pos: positive, neg: negative. NC: not computable. Significant differences are shown in bold. | | | | | |

**Supplementary Figure 1. Box plots for mean methylation levels of the host genes *EPB41L3* (CpG 438, 427, 425) and viral HPV16 L1 gene (CpG 6367, 6389) and the S5-Classifier (Reference) according to clinical outcome.** The lower and upper ends of the boxes indicate the 25% and the 75% values. Bars correspond to the 95% distribution interval with outlying observations indicated as individual dots. Pairwise comparison of the groups Progress (CIN3+) vs Regress (<CIN1) are shown in the graph with significance levels provided by the Mann Whitney test. Other significant differences (p≤0.05) were seen with the following group comparisons: Regress/Persist vs Progress (*EPB41L3* and S5-classifier) and Regress vs Persist/Progress (S5-classifier). The Cuzick test for trend among the individual clinical outcome groups were: 0.039 for *EPB41L3*; 0.244 for HPV16L1 and 0.001 for the S5-Classifier.


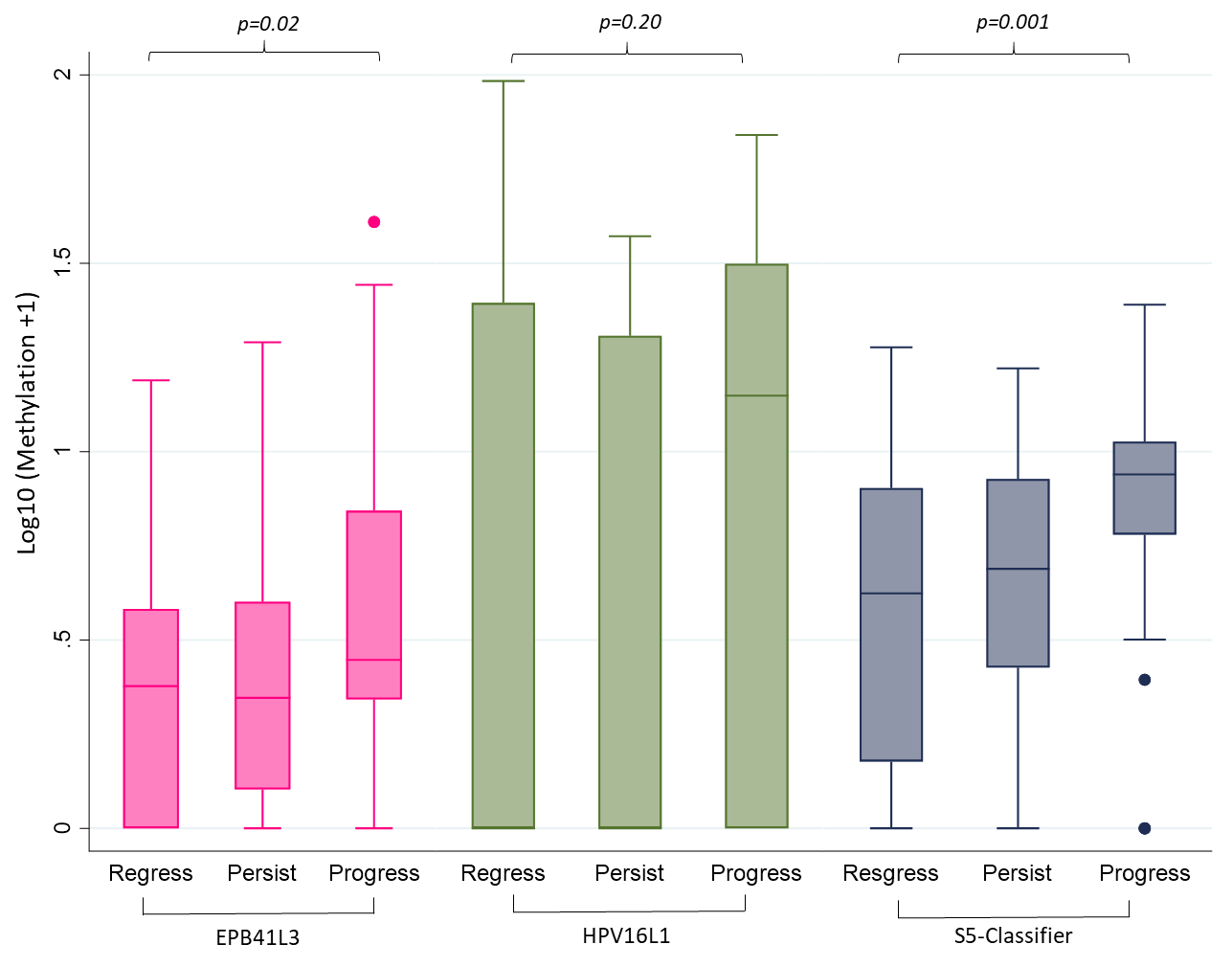


**Supplementary Figure 2. Receiver-operating characteristic (ROC) curve for the performance of methylation biomarkers of S5-classifier (green line), host gene *EPB41L3* (blue dotted line) and HPV16 L1 gene (maroon dash line) in the clinical outcome category of a) persistence vs. progression and b) regression vs. persistence/progression.** These ROC curves are showing different clinical outcome grouping than the main Figure 3. We can note that depending on the clinical outcome grouping the area under the ROC curve (AUC) can change substantially and these curves shown here may not be the optimal grouping for the methylation classifier.


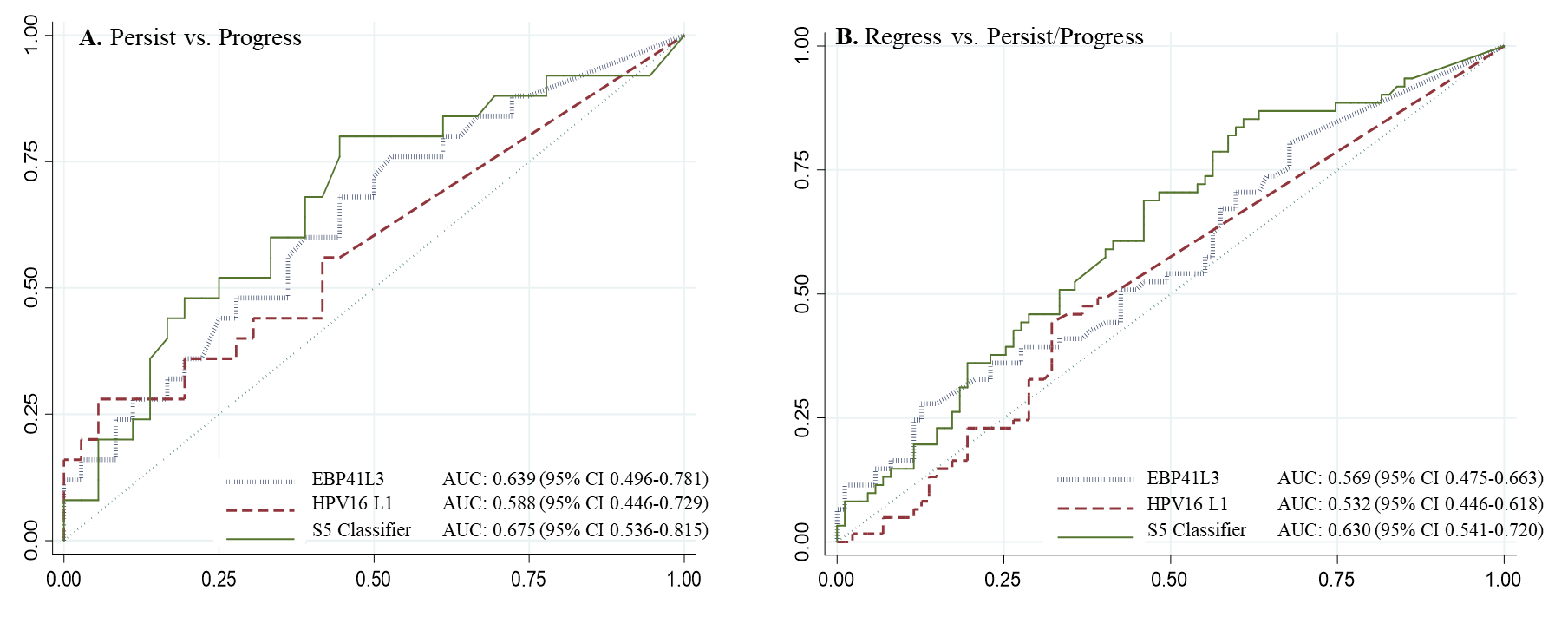


Abbreviations: AUC: Area Under the ROC Curve
